# Continuous-time quantum walk based centrality testing on weighted graphs

**DOI:** 10.1038/s41598-022-09915-1

**Published:** 2022-04-09

**Authors:** Yang Wang, Shichuan Xue, Junjie Wu, Ping Xu

**Affiliations:** Institute for Quantum Information & State Key Laboratory of High Performance Computing, College of Computer Science and Technology, National University of Defense Technology, Changsha, 410073 China

**Keywords:** Quantum information, Quantum simulation

## Abstract

Centrality measure is an essential tool in network analysis and widely used in the domain of computer science, biology and sociology. Taking advantage of the speedup offered by quantum computation, various quantum centrality measures have been proposed. However, few work of quantum centrality involves weighted graphs, while the weight of edges should be considered in certain real-world networks. In this work, we extend the centrality measure based on continuous-time quantum walk to weighted graphs. We testify the feasibility and reliability of this quantum centrality using an ensemble of 41,675 graphs with various topologies and comparing with the eigenvector centrality measure. The average Vigna’s correlation index of all the tested graphs with all edge weights in [1, 10] is as high as 0.967, indicating the pretty good consistency of rankings by the continuous-time quantum walk centrality and the eigenvector centrality. The intuitive consistency of the top-ranked vertices given by this quantum centrality measure and classical centrality measures is also demonstrated on large-scale weighted graphs. Moreover, the range of the continuous-time quantum walk centrality values is much bigger than that of classical centralities, which exhibits better distinguishing ability to pick the important vertices from the ones with less importance. All these results show that the centrality measure based on continuous-time quantum walk still works well on weighted graphs.

## Introduction

Random walks, as a fundamental tool, have been widely used in the fields of economics, computer science and natural sciences. Based on quantum mechanics, quantum walks are proposed as the quantum analog of classical random walks^1–4^. Both discrete-time^1,3,4^ and continuous-time^2^ versions are introduced corresponding to their classical counterpart. Benefited from the superposition of quantum systems, quantum walks have fundamentally different behavior compared to classical random walks, and become an essential tool in quantum computation and quantum algorithms. Childs proves that universal computation can be implemented by continuous-time quantum walks (CTQWs)^5^, and Lovett et al. present a version on discrete-time quantum walks (DTQWs)^6^. Moreover, quantum walks provide efficient quantum algorithms in a variety of scientific disciplines, such as search algorithm^7–9^, element distinctness^10,11^ and graph isomorphism^12,13^.

Vertex centrality ranking algorithm is another promising application of quantum walks. Vertex centrality is an integral tool in graph theory and network analysis, quantifying the importance of each node. The higher of the centrality measures, the more important of the corresponding nodes in the network. Centrality analysis has been widely used in ranking the webpages on the Internet^14,15^, identifying the most influential people in social networks^16^, finding out critical infrastructure nodes in urban networks^17^, and searching for super spreaders of infectious diseases^18^. There are several classical centrality measures including degree centrality, closeness centrality, betweenness centrality, eigenvector centrality and PageRank centrality. In general, distinct centrality measures underline different characteristics of the network, and are used in different scenarios.

As quantum walk outperforms its classical counterpart in many algorithmic applications^19,20^, various centrality algorithms based on quantum walks have been proposed. Quantum PageRank algorithm is proposed by Paparo and Martin-Delgado using Szegedy’s quantum walks (SQWs)^21^. Compared to the classical algorithm, the quantum PageRank algorithm is more sensitive, i.e., capable to highlight the secondary hubs and resolve the degeneracy of low lying nodes^22^. The SQW allows unitary evolution on directed and weighted graphs, but the Hilbert space required is $N^2$-dimension for a graph with *N* nodes. To physically implement quantum centrality in a smaller state space, Izaac et al. propose a quantum centrality measure based on CTQW. This CTQW-based centrality measure using a significantly smaller Hilbert space compared with the quantum PageRank algorithm, while taking advantage of the quantum speedup compared with the continuous-time random walk^23^. The CTQW-based centrality is limited to undirected network structures in the original study. The following works extend the CTQW-based centrality to directed networks^24^ and experimentally demonstrate the quantum centrality ranking on directed graphs^25,26^.

Except for the direction of connections in networks, the weight of connections is also an essential factor in real-world networks. For instance, Radicchi et al. define the weighted citation network between authors, and use it to analyze the scientific impact of physicists^27^. Another example is to identify the super spreaders in an outbreak of infectious disease^18^. The close contacts and accidental meetings usually face different infection risks, and should be marked differently in contact tracing to better control the spread of the virus. These two examples can be abstracted to centrality ranking problem, where the prominent physicists in citation networks and the super spreaders of infectious diseases are denoted by higher centrality measure. The weight or the number of connections between two nodes effect the centrality ranking in these cases.

In this work, we extend the CTQW-based centrality measure to the weighted graph for the first time. We test an ensemble of weighted graphs with various topologies to validate the feasibility and reliability of this quantum centrality. The correlation coefficients between the rankings given by CTQW-based centrality and eigenvector centrality are pretty high for all the tested graphs, and intuitive consistency of the top-ranked vertices given by this quantum centrality measure and classical centrality measures is also demonstrated on large-scale weighted graphs. What’s more, we also find the advantage of the CTQW-based centrality in distinguishing the important vertices from the ones with less importance.

## Results

The time evolution of the CTQW is described by the time-independent Hamiltonian, which is determined by the underlying network structure. Specifically for an undirected graph *G* with *n* vertices, a quantum walker evolves in the walking space spanned by $\{|1\rangle ,|2\rangle , \ldots ,|n\rangle \}$, which is an orthogonal basis corresponding to *n* vertices. The Schrödinger equation governs the evolution of CTQW on graph *G*^2,22^:

1 $$\begin{aligned} i\hbar \frac{d}{dt}|\psi (t)\rangle =H|\psi (t)\rangle , \end{aligned}$$

where the Hamiltonian is the adjacent matrix ($H=A$) and

2 $$\begin{aligned} A_{ij}= {\left\{ \begin{array}{ll} w_{ij}&{} \mathrm{\ for\ node}\ i\ \mathrm{and}\ j\ \mathrm{\ connected\ with\ weight\ }w_{ij},\\ 0&{} \mathrm{\ for\ node}\ i\ \mathrm{and}\ j\ \mathrm{\ not\ connected}. \end{array}\right. } \end{aligned}$$

To extend the CTQW-based centrality measure to weighted graphs, it should be noted that the elements in the adjacent matrix *A* denote the weight of each edge. Solving the Schrödinger equation with $\hbar =1$, we get the state of the quantum walker at time *t*:

3 $$\begin{aligned} |\psi (t)\rangle&= e^{-i H t}|\psi (0)\rangle =\sum _{i=1}^{n} \alpha _{i}(t)|i\rangle . \end{aligned}$$

$|\psi (t)\rangle$ is a superposition state in the walking space, where the probability amplitude on node *i* at time *t* is $\alpha _{i}(t)=\langle {i|\psi (t)}\rangle$. The probability of the walker at node *i* is $P_{i}(t)=\left| \alpha _{i}(t)\right| ^{2}$.

For the centrality measure based on CTQWs, the evolution starts from the initial state $|\psi (0)\rangle =\frac{1}{\sqrt{n}} \sum _{i=1}^{n} |i\rangle$. The quantum walker propagates on graph *G* following Eq. (3). The CTQW-based centrality is calculated by the long-time average of the walker located at each vertex^22^:

4 $$\begin{aligned} c_i^{(CTQW)}=\bar{P_{i}}=\lim _{t \rightarrow \infty } \frac{1}{t} \int _{0}^{t} \left| \langle {i|\psi (t)}\rangle \right| ^{2} d t. \end{aligned}$$

**Figure 1:**
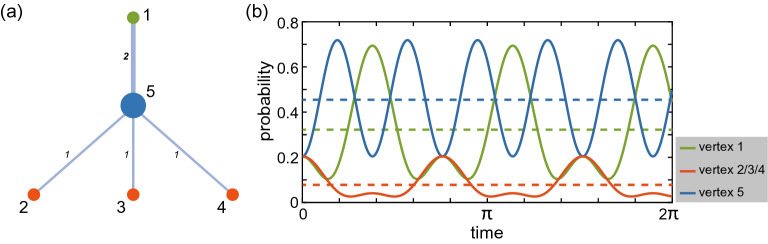
(**a**) A typical 5-node network. (**b**) Probability distribution on each vertex *i* along the evolution time *t*, where the three red vertices show identical behaviours. The dotted line denotes the average probability, thus the CTQW-based centrality of each vertex $c^{(CTQW)}_i$.

In Fig. 1, we use a simple example to demonstrate the numerical simulation of the CTQW-based centrality on a weighted graph. Figure 1a gives a typical 5-node network, and the weights are marked on each edge. The probability on each vertex as a function of evolution time is shown in Fig. 1b. The dotted line denotes the average probability and is the CTQW-based centrality of each vertex. According to these centrality values, the ranks of node 1 to node 5 are 2, 3, 3, 3, 1 respectively, which are the same as the order given by eigenvector centrality. The previous works show that the CTQW-based centrality correlates well with the eigenvector centrality, and works excellently as a centrality measure on unweighted graphs^23,24^. The simple example in Fig. 1 shows the possibility that the CTQW-based centrality may still work on weighted graphs. We now generalize the CTQW-based centrality to the weighted graphs and study the performance of this quantum centrality measure.

**Table 1:** The average correlation coefficients for graphs with different topologies.

Graphs	*n*	Group size	$\tau ^{w=2}_{Kendall}$	$\tau ^{w=2}_{Vigna}$	$\tau ^{w_{(i,j)}\in [1,10]}_{Kendall}$	$\tau ^{w_{(i,j)}\in [1,10]}_{Vigna}$
Connected	4	6	$1.000 \pm 0.000$	$1.000 \pm 0.000$	$1.000 \pm 0.000$	$1.000 \pm 0.000$
	5	21	$0.983 \pm 0.003$	$0.982 \pm 0.003$	$0.943 \pm 0.013$	$0.966 \pm 0.005$
	6	112	$0.963 \pm 0.009$	$0.975 \pm 0.004$	$0.974 \pm 0.004$	$0.983 \pm 0.002$
Planar	7	646	$0.940 \pm 0.009$	$0.959 \pm 0.005$	$0.933 \pm 0.008$	$0.954 \pm 0.004$
	8	5974	$0.932 \pm 0.008$	$0.957 \pm 0.004$	$0.927 \pm 0.007$	$0.954 \pm 0.004$
Eulerian	8	184	$0.940 \pm 0.010$	$0.958 \pm 0.006$	$0.947 \pm 0.008$	$0.966 \pm 0.003$
	9	1782	$0.943 \pm 0.007$	$0.961 \pm 0.004$	$0.947 \pm 0.004$	$0.965 \pm 0.002$
Vertex critical	10	5284	$0.966 \pm 0.002$	$0.978 \pm 0.001$	$0.963 \pm 0.002$	$0.976 \pm 0.001$
Edge critical	12	17431	$0.966 \pm 0.001$	$0.978 \pm 0.001$	$0.967 \pm 0.001$	$0.979 \pm 0.001$
Self-complementary	12	720	$0.963 \pm 0.002$	$0.973 \pm 0.001$	$0.959 \pm 0.002$	$0.972 \pm 0.001$
	13	5600	$0.956 \pm 0.002$	$0.969 \pm 0.002$	$0.962 \pm 0.001$	$0.974 \pm 0.001$
Cubic planar	16	233	$0.613 \pm 0.054$	$0.822 \pm 0.013$	$0.828 \pm 0.026$	$0.911 \pm 0.007$
	18	1249	$0.542 \pm 0.063$	$0.790 \pm 0.015$	$0.816 \pm 0.027$	$0.905 \pm 0.008$
Hypo-Hamiltonian	26	2033	$0.835 \pm 0.023$	$0.930 \pm 0.004$	$0.852 \pm 0.013$	$0.929 \pm 0.003$
*ER*(100, 0.3)	100	200	$0.991 \pm 0.000$	$0.995 \pm 0.000$	$0.989 \pm 0.000$	$0.994 \pm 0.000$
*SF*(100, 2)	100	200	$0.614 \pm 0.004$	$0.842 \pm 0.001$	$0.576 \pm 0.005$	$0.821 \pm 0.002$

The superscript $w=2$ denotes only one weighed edge $w=2$, and $w_{(i,j)} \in [1,10]$ denotes the weight of arbitrary edge in [1, 10].

**Figure 2:**
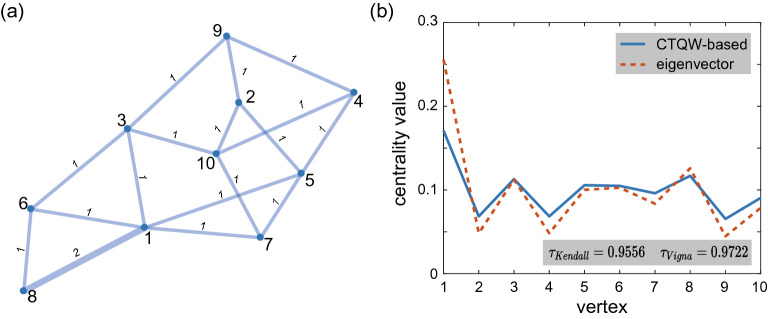
(**a**) A randomly generated Erdős-Rényi graph *ER*(10, 0.3) with an additional weight $w=2$ added on a random edge. (**b**) The eigenvector centralities (dotted red line) and CTQW-based centralities (solid blue line) for vertices of the graph in (**a**).

To validate our proposal’s feasibility, we conduct statistical analysis over an ensemble of randomly generated weighted graphs, including connected, planar, Eulerian, vertex critical, edge critical, self-complementary, cubic planar, hypo-Hamiltonian, Erdős-Rényi and scale-free graphs, 41,675 graphs in total. The original graphs are undirected and unweighted. So we add a weight $w=2$ on a randomly chosen edge to all graphs in each group. The tested graphs are listed in Table 1. Figure 2 intuitively shows the correlation of the eigenvector and CTQW-based centralities in the ranking problem. Referring to the time analysis of the centrality based on DTQWs^28^, all the CTQW-based centralities of weighted graphs in this paper are calculated over the same timescale, i.e., $t=20\pi$ instead of $t \rightarrow \infty$. To quantitatively evaluate the agreement between the rankings by eigenvector and CTQW-based centralities, we employ Kendall’s $\tau$ correlation coefficient^29^ (see “Methods” for the calculation of $\tau _{Kendall}$). $\tau _{Kendall}$ takes values from $-1$ to 1, wherein $\tau _{Kendall} = 1$ denotes the same ranking orders by different centrality measures. Two rankings with $\tau _{Kendall}$ at or above 0.9 are considered effectively equivalent^30,31^, at least empirically^32,33^. It can be seen that among all generated weighted graphs listed in Table 1, the $\tau ^{w=2}_{Kendall}$ between the eigenvector and the CTQW-based centrality measures are pretty high except for the cubic planar and scale-free graphs.

**Figure 3:**
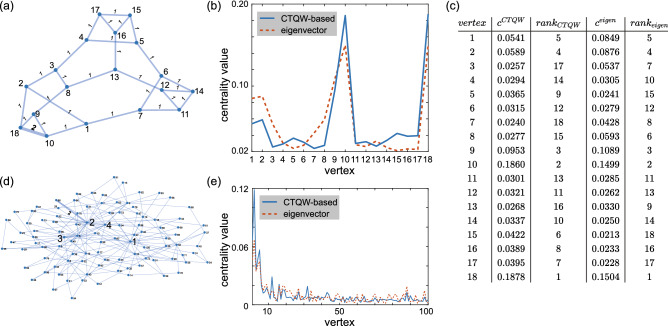
(**a**) The cubic planar graph with the minimal $\tau ^{w=2}_{Kendall}$ of all the tested cubic planar graphs. (**b**) The eigenvector centralities (dotted red line) and CTQW-based centralities (solid blue line) for vertices of the graph in (**a**). (**c**) The ranking orders for the vertices of the graph in (**a**) by the eigenvector centrality measure and CTQW-based centrality measure. (**d**) The scale-free network *SF*(100, 2) with the minimal $\tau ^{w=2}_{Kendall}$ of all the tested *SF*(100, 2) with only one edge weighted 2. **(e)** The eigenvector centralities (dotted red line) and CTQW-based centralities (solid blue line) for vertices of the graph in (**d**).

To find the reason causing the imperfect correlation, the eigenvector and the CTQW-based centralities of the cubic planar graph and the scale-free graph with the minimum $\tau _{Kendall}$ are shown in Fig. 3. We can see that the correlation is lowered by the discordances in low-lying vertex centralities while the important vertices with large centralies ranked the same by the eigenvector and the CTQW-based centrality measures. However, the low-lying centrality usually give little information and we care more about the top-ranked vertexes in most cases. The calculation of $\tau _{Kendall}$ does not make any distinctions and equally penalizes discordances both at high and low rankings. There have been many researches to cover this flaw of $\tau _{Kendall}$ in certain applications^31,34–38^, and we further employ a weighted variant of Kendall’s correlation coefficient introduced by Vigna^38^, $\tau _{Vigna}$, to evaluate the agreement between the rankings by eigenvector and CTQW-based centralities. The Vigna’s rank correlation coefficient gives more weight to the discordances at high rankings, whose usefulness has been validate on social networks and web graphs^38^. The average $\tau _{Vigna}$ for each graph set is listed in Table 1. The correlation coefficient values increase especially for the cubic planar and scale-free graphs as expected. The average $\tau _{Vigna}$ of all the tested 41,675 graphs is 0.963, which indicates a consistent ranking order with eigenvector centrality achieved on large-scale test. Hence, it is reasonable to utilize our CTQW-based centrality measure to solve the centrality problem on weighted graphs.

**Figure 4:**
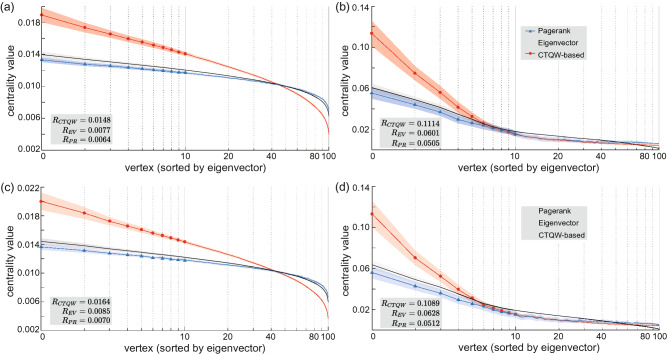
The average CTQW-based, eigenvector and PageRank centralities for vertices over an ensemble of 200 Erdős-Rényi graphs *ER*(100, 0.3) with only one weighted edge $w=2$ (**a**), scale-free networks *SF*(100, 2) with only one weighted edge $w=2$ (**b**), *ER*(100, 0.3) with all edges weighed in [1, 10] (**c**), and *SF*(100, 2) with all edges weighed in [1, 10] (**d**), The vertices are sorted by the eigenvector centrality measure and the shaded areas represent one standard deviation from the average centralities among 200 graphs. It is obvious that the range of CTQW-based centrality measure $R_{CTQW}$ is much bigger than other measures.

The above analysis based on correlation coefficients has shown the excellent consistency of the rankings by CTQW-based centrality and eigenvector centrality. However, from the correlation coefficients we are still not sure if the top-ranked vertices hold the exactly same ranking order. So it is proper to give an intuitive demonstration of the consistence details. As most cases especially concern the top ranks, we pay more attention to the top-ranked vertices. Figure 4 intuitively demonstrates the consistence of rankings by PageRank, eigenvector and CTQW-based centralities on ensembles of the large-scale weighted graphs. Concretely, we consider two of the most paradigmatic network topologies: Erdős-Rényi graphs^39^ and scale-free networks^40^. An Erdős-Rényi graph denoted by *ER*(*n*, *p*) is comprised of *n* vertices with edges randomly distributed following the Bernoulli distribution with probability *p*. For such a network, the vertex degree distribution *P*(*k*) (the fraction of vertices with degree *k*) follows binomial form, i.e., most vertices have a degree close to the mean number of connections, $n\cdot p$. A scale-free network *SF*(*n*, *m*) is generated by Barabási-Albert algorithm^41^ with *n* vertices and the probability of *k*-degree vertex $p(k) \propto \frac{1}{k^m}$. In a scale-free network, most vertices have only a few connections with others, and a few vertices are connected with a large number of other vertices, which are called hub vertices. We take the eigenvector centrality measure as the benchmark and sort the vertices by the eigenvector centrality values. The average CTQW-based centrality, eigenvector centrality and PageRank centrality of each vertices over the ensemble of *ER*(100, 0.3) and the ensemble of *SF*(100, 2) are shown in Fig. 4a,b respectively. The 100 vertices are ranked by their eigenvector centralities, so the eigenvector centrality (grey dotted line) decreases monotonically. It is obvious that the vertices with the top 10 eigenvector centralities are also top of the CTQW-based and PageRank centrality rankings, and the Pagerank (blue triangle) and the CTQW-based (red circle) centrality values also present monotone decreasing tendency, which means that the ranking orders are the same as the eigenvector centrality. Moreover, Fig. 4a,b show distinctive centrality distributions corresponding to the topological features of Erdős-Rényi and scale-free networks. The centrality values of the most important vertices in scale-free networks are almost one order of magnitude higher than those in Erdős-Rényi graphs, i.e. the few hub vertices in scale-free network show a more dominant role in the whole network. These features indicate the reliability of the CTQW-based centrality on weighted graphs in ranking the top-ranked vertices.

It is worth noting that the range of the CTQW-based centrality is larger than the eigenvector centrality and PageRank centrality, which means the better distinction of the vertex centrality ranks. We use the range $R=c_{max}-c_{min}$ to evaluate the distribution of the centrality values. $R_{CTQW}$ for CTQW-based centrality measure is almost twice the $R_{EV}$ and $R_{PR}$ for eigenvector and Pagerank centrality measures on the randomly generated Erdős-Rényi and scale-free graphs. It is reasonable to utilize the distinguishing ability of CTQW-based centrality measure to pick the important vertices from the ones with less importance.

**Figure 5:**
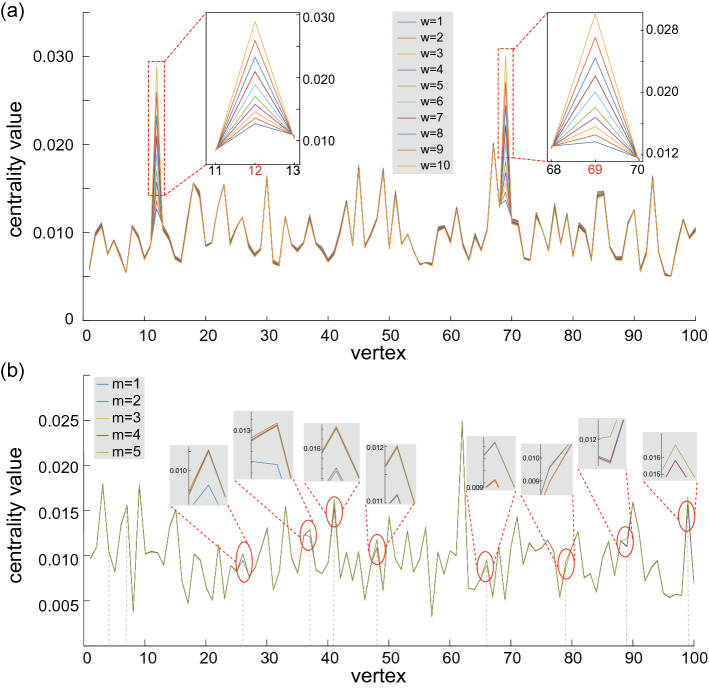
(**a**) CTQW-based centralities on an *ER*(100, 0.3) by traversing weight of an edge from 1 to 10. The randomly chosen edge connects vertex 12 and 69. The inset graphs show details of centrality changes near vertex 12 and 69. (**b**) More weighted edges on Erdős-Rényi graphs *ER*(100, 0.3). The number of weighted edges, *m*, ranges from 1 to 5, and the weight $w=2$ is added to the edge (4, 7), (26, 37), (41, 48), (66, 79) and (89, 99) in succession. The vertical dotted lines show the endpoint vertices of edges with additional weights. The inset graphs give details of centrality changes near these endpoint vertices.

It has been shown that the CTQW-based centrality measure works well on weighted graphs with $w=2$ added on one randomly-chosen edge. The weighted graphs must be more complicated in the real world, so we further investigate the CTQW-based centrality by varying weight and choosing more weighted edges. First, we generate an Erdős-Rényi graph *ER*(100, 0.3) and assign different weights from 1 to 10 to the edge connecting vertex 12 and 69. Then we conduct the numerical simulations to observe the changes of CTQW-based centrality values for each vertex. The results are shown in Fig. 5a, where different weight cases correspond to lines of different colors. It can be seen that there is little influences on vertices other than the endpoint vertex 12 and 69 of the randomly chosen edge with additional weights. From the insets of Fig. 5a, it is clear that the centrality values rise up as the weight increasing. Besides varying the weight of a certain edge, we also considering the case of multiple weighted edges. For the *ER*(100, 0.3), we randomly choose 5 edges and add the weight $w=2$ to these edges in succession. The calculated CTQW-based centralities are shown in Fig. 5b, which also indicates that the weight mainly influence the centralities of the corresponding endpoints. This conclusion is in line with the intuitive cognition.

Based on the above analysis, we finally test an ensemble of graphs whose each edge is given a random weight $w \in [1,10]$. The original graphs are the same as those listed in Table 1, but the weight of each edge is newly generated. Numerical analysis based on $\tau _{Kendall}$ and $\tau _{Vigna}$ is conducted on these new weighted graphs, and the results are shown in Table 1. It is clear that the correlation coefficients are still reasonably high with ${\bar{\tau }}^{w_{(i,j)}\in [1,10]}_{Vigna}=0.967$, which indicates the excellent consistency of the rankings by CTQW-based centrality and eigenvector centrality. Figure 4c,d further intuitively demonstrate the rankings by PageRank, eigenvector and CTQW-based centralities on the new weighted *ER*(100, 0.3) and *SF*(100, 2) graphs respectively. The reliability in ranking the top-ranked vertices and the distinguishing ability of CTQW-based centrality still holds in these large-scale graphs with all random-weighted edges. In conclusion, it is reasonable to extend the CTQW-based centrality to the weighted graphs.

## Discussion

In summary, we extend the CTQW-based centrality to weighted graphs for the first time, which expands the use of CTQW-based centrality measure to more realistic applications. Based on the numerical analysis on various weighted graphs, we testify the feasibility and reliability of this quantum centrality. The correlation coefficients between the rankings by CTQW-based centrality and eigenvector centrality are pretty high, and the ranks of top-ranked vertices given by this quantum centrality and the classical centralities consist well according to the intuitive demonstrations. Furthermore, we find that the CTQW-based centrality measure show better distinguishing ability to pick the important vertices from the ones with less importance. All the excellent results are obtained using an instance simulation time for CTQW-based centralities. For the precise analysis of this quantum centrality efficiency, further investigation is needed to compare with the classical algorithms.

## Methods

### Kendall’s $\tau$ correlation

Suppose $X = \langle x_{1}, x_{2}, \ldots , x_{n} \rangle$ are eigenvector centralities of vertices in graph *G*, and $Y = \langle y_{1}, y_{2}, \ldots , y_{n} \rangle$ are CTQW-based centralities. The subscript from 1 to *n* identifies the vertex. The Kendall’s $\tau$ coefficient is used to measure the agreement between the rankings given by these centrality measures, i.e.,

5 $$\begin{aligned} \tau _{Kendall}&=\frac{\sum _{i<j} sgn\left( x_i-x_j\right) sgn\left( y_i-y_j\right) }{\sqrt{\sum _{i<j} sgn\left( x_i-x_j\right) sgn\left( x_i-x_j\right) }\sqrt{\sum _{i<j} sgn\left( y_i-y_j\right) sgn\left( y_i-y_j\right) }}\nonumber \\ &=\frac{\sum _{i<j} sgn\left( x_i-x_j\right) sgn\left( y_i-y_j\right) }{\sqrt{n(n-1)/2-t_X}\sqrt{n(n-1)/2-t_Y}}, \end{aligned}$$

6 $$\begin{aligned} sgn\left( r\right)&= \left\{ \begin{array}{ccc} 1 &{} \text{ if } r>0 \\ 0 &{} \text{ if } r=0 \\ -1 &{} \text{ if } r<0 \end{array}\right. , \end{aligned}$$

where $n(n-1)/2$ is the total number of pairs (*i*, *j*) with $i<j$ and $t_X\ (t_Y)$ is the number of tied pairs in the ranking $X\ (Y)$. For arbitrary pair of vertices $\left( i, j\right)$, the two centrality measures are said to be concordant if $sgn\left( x_{i}-x_{j}\right) sgn\left( y_{i}-y_{j}\right) >0$, and discordant if $sgn\left( x_{i}-x_{j}\right) sgn\left( y_{i}-y_{j}\right) <0$. A tie reflects the inability of the centrality measure to decide which item should be ranked first, and the tied pair with $\left( x_{i}-x_{j}\right) =0$ or $\left( y_{i}-y_{j}\right) =0$ can be considered neither concordant nor discordant. The coefficient $\tau _{Kendall} = 1$ if and only if there is a perfect correspondence between the rankings of vertices in *G* with reference to the different centrality measures, and $\tau _{Kendall} = -1$ indicates that the rankings are exactly inverted. Therefore, the coefficient $\tau _{Kendall}$ closer to 1 means that the CTQW-based centrality is more consistent with eigenvector centrality measure, and is feasible as a evaluation criterion.

### The weighted correlation index

$\tau _{Vigna}$. Vigna’s Correlation index $\tau _{Vigna}$ for rankings extends Kendall’s definition taking into account weights of concordances and discordances between vertices with different ranks in the presence of ties. The weight function used in this paper is

7 $$\begin{aligned} w(i,j)=\frac{1}{\rho (i)}+\frac{1}{\rho (j)}. \end{aligned}$$

$\rho (i)$ is the ranking function associating each vertex with a rank. In this paper, $\rho _{X,Y}(i)$ and $\rho _{Y,X}(i)$ give a unique rank to each vertex. We denote different ranking functions by distinct subscripts. $\rho _{X,Y}(i)$ is defined by ranking vertices in descending order with respect to *X* and then *Y*. For the vertices tied both in *X* and *Y*, they can be placed in any order. The function $\rho _{Y, X}(i)$ is defined analogously. The weighted correlation index is calculated by

8 $$\begin{aligned} \begin{aligned} \tau _{Vigna} & = \frac{1}{2} \left(\frac{\sum _{i<j} sgn\left( x_i-x_j\right) sgn\left( y_i-y_j\right) w_{X, Y}(i,j)}{\sqrt{\sum _{i<j} sgn\left( x_i-x_j\right) w_{X, Y}(i,j)}\sqrt{\sum _{i<j} sgn\left( y_i-y_j\right) w_{X, Y}(i,j)}} \right. \\ & \quad \left. +\frac{\sum _{i<j} sgn\left( x_i-x_j\right) sgn\left( y_i-y_j\right) w_{Y, X}(i,j)}{\sqrt{\sum _{i<j} sgn\left( x_i-x_j\right) w_{Y, X}(i,j)}\sqrt{\sum _{i<j} sgn\left( y_i-y_j\right) w_{Y, X}(i,j)}}\right). \end{aligned} \end{aligned}$$

The Kendall’s $\tau$ coefficient is lowered by the discordances of the vertices with large ranks comparing to Vigna’s weighted correlation index.

## Data Availability

The datasets generated during and/or analysed during the current study are available from the corresponding author on reasonable request.

## References

[1] Aharonov Y, Davidovich L, Zagury N (1993). Quantum random walks. Phys. Rev. A.

[2] Farhi E, Gutmann S (1998). Quantum computation and decision trees. Phys. Rev. A.

[3] Ambainis, A., Bach, E., Nayak, A., Vishwanath, A. & Watrous, J. One-dimensional quantum walks. In *Proceedings of the 33rd Annual ACM Symposium on Theory of Computing*, 37–49 (2001).

[4] Aharonov, D., Ambainis, A., Kempe, J. & Vazirani, U. Quantum walks on graphs. In *Proceedings of the 33rd Annual ACM Symposium on Theory of Computing*, 50–59 (2001).

[5] Childs AM (2009). Universal computation by quantum walk. Phys. Rev. Lett..

[6] Lovett NB, Cooper S, Everitt M, Trevers M, Kendon V (2010). Universal quantum computation using the discrete-time quantum walk. Phys. Rev. A.

[7] Shenvi N, Kempe J, Whaley KB (2003). Quantum random-walk search algorithm. Phys. Rev. A.

[8] Childs AM, Goldstone J (2004). Spatial search by quantum walk. Phys. Rev. A.

[9] Tulsi A (2008). Faster quantum-walk algorithm for the two-dimensional spatial search. Phys. Rev. A.

[10] Ambainis A (2007). Quantum walk algorithm for element distinctness. SIAM J. Comput..

[11] Magniez F, Nayak A, Roland J, Santha M (2011). Search via quantum walk. SIAM J. Comput..

[12] Douglas BL, Wang JB (2008). A classical approach to the graph isomorphism problem using quantum walks. J. Phys. A Math. Theor..

[13] Berry SD, Wang JB (2011). Two-particle quantum walks: Entanglement and graph isomorphism testing. Phys. Rev. A.

[14] Brin S, Page L (1998). The anatomy of a large-scale hypertextual web search engine. Comput. Netw. ISDN Syst..

[15] Brin S (1998). The Pagerank citation ranking: Bringing order to the Web. Proc. ASIS.

[16] Shetty, J. & Adibi, J. Discovering important nodes through graph entropy the case of Enron email database. In *Proceedings of the 3rd International Workshop on Link Discovery*, 74–81 (2005).

[17] Shen Y, Nguyen NP, Xuan Y, Thai MT (2012). On the discovery of critical links and nodes for assessing network vulnerability. IEEE/ACM Trans. Netw..

[18] Dekker, A. Network centrality and super-spreaders in infectious disease epidemiology. In *20th International Congress on Modelling and Simulation (MODSIM2013)* (2013).

[19] Ambainis A (2003). Quantum walks and their algorithmic applications. Int. J. Quant. Inf..

[20] Childs, A. M. *et al.* Exponential algorithmic speedup by a quantum walk. In *Proceedings of the 35th Annual ACM Symposium on Theory of Computing*, 59–68 (2003).

[21] Paparo GD, Martin-Delgado M (2012). Google in a quantum network. Sci. Rep..

[22] Paparo GD, Müller M, Comellas F, Martin-Delgado MA (2013). Quantum google in a complex network. Sci. Rep..

[23] Izaac JA (2017). Centrality measure based on continuous-time quantum walks and experimental realization. Phys. Rev. A.

[24] Izaac J, Wang J, Abbott P, Ma X (2017). Quantum centrality testing on directed graphs via PT-symmetric quantum walks. Phys. Rev. A.

[25] Wang K (2020). Experimental realization of continuous-time quantum walks on directed graphs and their application in Pagerank. Optica.

[26] Wu T (2020). Experimental parity-time symmetric quantum walks for centrality ranking on directed graphs. Phys. Rev. Lett..

[27] Radicchi F, Fortunato S, Markines B, Vespignani A (2009). Diffusion of scientific credits and the ranking of scientists. Phys. Rev. E.

[28] Loke T, Tang JW, Rodriguez J, Small M, Wang JB (2017). Comparing classical and quantum Pageranks. Quant. Inf. Process..

[29] Kendall MG (1938). A new measure of rank correlation. Biometrika.

[30] Voorhees, E. M. Evaluation by highly relevant documents. In *Proceedings of the 24th Annual International ACM SIGIR Conference on Research and Development in Information Retrieval*, 74–82 (2001).

[31] Yilmaz, E., Aslam, J. A. & Robertson, S. A new rank correlation coefficient for information retrieval. In *Proceedings of the 31st Annual International ACM SIGIR Conference on Research and Development in Information Retrieval*, 587–594 (2008).

[32] Sanderson, M. & Joho, H. Forming test collections with no system pooling. In *Proceedings of the 27th Annual International ACM SIGIR Conference on Research and Development in Information Retrieval*, 33–40 (2004).

[33] Carterette, B. & Allan, J. Incremental test collections. In *Proceedings of the 14th ACM International Conference on Information and Knowledge Management*, 680–687 (2005).

[34] Shieh GS (1998). A weighted Kendall’s tau statistic. Stat. Probab. Lett..

[35] Wu, S. & Crestani, F. Methods for ranking information retrieval systems without relevance judgments. In *Proceedings of the 2003 ACM Symposium on Applied Computing*, 811–816 (2003).

[36] Voorhees, E. M. Overview of the TREC 2004 robust retrieval track. In *Proceedings of the 13th Text REtrieval Conference (TREC2004)*, 13 (2004).

[37] Haveliwala, T. H., Gionis, A., Klein, D. & Indyk, P. Evaluating strategies for similarity search on the Web. In *Proceedings of the 11th International Conference on World Wide Web*, 432–442 (2002).

[38] Vigna, S. A weighted correlation index for rankings with ties. In *Proceedings of the 24th International Conference on World Wide Web*, 1166–1176 (2015).

[39] Erdos P, Rényi A (1960). On the evolution of random graphs. Publ. Math. Inst. Hung. Acad. Sci.

[40] Barabási A-L, Albert R (1999). Emergence of scaling in random networks. Science.

[41] Albert R, Barabási A-L (2002). Statistical mechanics of complex networks. Rev. Mod. Phys..

